# Danggui-Shaoyao-San Improves Learning and Memory in Female SAMP8 via Modulation of Estradiol

**DOI:** 10.1155/2014/327294

**Published:** 2014-03-16

**Authors:** Yan Huang, Zeng-yao Hu, Hui Yuan, Lei Shu, Gang Liu, Shan-yi Qiao, Lei Sun, Wen-xia Zhou, Yong-xiang Zhang

**Affiliations:** Beijing Institute of Pharmacology and Toxicology, Tai Ping Road 27, Beijing 100850, China

## Abstract

Previous studies showed that Danggui-Shaoyao-San (DSS), a traditional Chinese medicinal prescription, could alleviate cognitive dysfunction of Alzheimer's disease (AD) patients. However, the mechanisms remain unclear; we have now examined the effect of DSS on SAMP8 and elucidated the possible mechanism. Animals were treated with DSS for 2 months, and step-down test and Morris water maze (MWM) test were used to evaluated cognitive abilities. The estradiol (E2), NO, and glycine in blood plasma or in hippocampus were detected to explore the possible mechanisms. The latency of SAMP8 in step-down test was shorter than that of age-matched SAMR1, and DSS increased the latency especially in female animals. In MWM test, we got similar results; SAMP8 spent more time to find the platform, and DSS decreased the time before finding the platform, with little effect on swim velocity, during the training sessions. During test session, DSS increased the time spent in target quadrant especially in female SAMP8. In female SAMP8, plasma E2, NO, and glycine were elevated in plasma or hippocampus tissue. In conclusion, DSS could ameliorate deterioration of cognition in SAMP8, especially in female animals. Increasing E2, NO, and glycine might contribute to the cognitive improvement effect of DSS in female SAMP8.

## 1. Introduction

Alzheimer's disease (AD) is the most prevalent neurodegenerative disorder of the central nervous system in old people [1]. Many studies have showed that women are more vulnerable to AD than men [2–4]. Furthermore, AD pathology [5–7] and AD-related cognitive decline [4, 5, 8–10] are greater in women than in men. Previous studies demonstrated that women with lower level of estrogen have higher risk for AD compared with age matched controls [11–13]. In animal models, experimental depletion of sex steroid hormones by ovariectomy (OVX) could increase A*β* accumulation, and E2 has preventive effects against OVX induced A*β* accumulation [13–17].

Danggui-Shaoyao-San (DSS), a traditional Chinese medicinal prescription, is used widely in oriental countries, such as China, Japan, and Korea [18–22]. DSS was initially recorded in “Synopsis of Prescriptions of the Golden Chamber,” which was compiled by Zhong-Jing Zhang during the Han dynasty. This prescription was traditionally used to relieve menorrhagia and other abdominal pains of women; modulation of estrogen is believed to be one of its mechanisms. DSS could increase the estrogen level in OVX rats [23] and stimulates estrogen production in vitro [24].

In the 1980s, the therapeutic effect of DSS on AD was first reported by researchers in Japan [25]. Many researchers try to unveil the underlying mechanisms of DSS on AD; modulation of cholinergic system, monoaminergic system, and neurotransmitters are believed to be some of the mechanisms [26–30]. In our previous study, we found DSS and one of its fractions (JD-30) have protective effects on some animal model for AD [31, 32]. Although we have made great progress in understanding how DSS affects AD, the mechanisms need further explorations.

Based on previous studies, we presume that modulation of estrogen is one of the mechanisms for DSS against AD. In this study, we used senescence-accelerated mouse prone 8 (SAMP8) as AD model to examine our hypothesis.

## 2. Materials and Methods

### 2.1. Preparation of DSS

DSS is composed of the following 6 raw herbs:* Angelica sinensis* (Oliv.) Diels (Umbelliferae),* Paeonia lactiflora* Pall. (Ranunculaceae),* Ligusticum chuanxiong* Hort. (Umbelliferae),* Poria cocos* (Schw.) Wolf (Polyporaceae),* Atractylodes macrocephala* Koidz. (Compositae), and* Alisma orientalis* (Sam.) Juzep. (Alismataceae). These materials purchased from Tongrentang Pharmaceutical Company (Beijing, China) were authenticated by Dr. Y. M. Zhao and Dr. Q. Y. Ma, both being botanists in the Department of Phytochemistry in our institute. The voucher specimens were deposited in the Department of Phytochemistry, Beijing Institute of Pharmacology and Toxicology.

The 6 raw herbs were mixed in the dry weight ratio of 3 : 16 : 8 : 4 : 4 : 8, and the mixture was left in 95% ethanol (1 : 5 w/v) overnight at room temperature and boiled twice for 2 h each time. After filtration and centrifugation, the extract was concentrated and referred to as DSS-A (10.36%, w/w). The residue was boiled with distilled water twice for 1 h each time and filtered to obtain the filtrate. The filtrate was concentrated and lyophilized to obtain the preparation referred to as DSS-W (13.84%, w/w). DSS-A and DSS-W were mixed and concentrated to 1 g/mL, known as DSS extract, and stored at 4°C.

### 2.2. Animals Groups and Drug Administration

Senescence-accelerated mouse resistant 1 (SAMR1) and SAMP8 mice were kindly provided by Dr. T. Takeda at Kyoto University, Japan. The mice were maintained in the Beijing Institute of Pharmacology and Toxicology under a 12 h light/12 h dark cycle at a constant temperature of 25 ± 1°C, with a humidity of 55 ± 5%, and were fed a standard rodent diet. They were allowed free access to water and food. The animal treatment, husbandry, and experimental protocols in this study were approved by the institute's Animal Care and Use Committee (IACUC) of the National Beijing Center for Drug Safety Evaluation and Research (NBCDSER).

Seven-month-old SAMP8 mice were separated into 4 groups at random, each group contains 20 mice (10 males and 10 females). DSS was administrated by intragastric at 1.6, 3.2, 4.8 g/kg body weight. Control group and the age-matched SAMR1 (10 males and 10 females) were given an equal volume of distilled water. Behavioral tests were performed 8 weeks after drug administration, and the drugs administration lasted until all tests were finished.

### 2.3. Step-Down Test

The tests were carried out between 8:00 and 12:00 AM. The apparatus was a 50 × 25 × 25 cm^3^ Plexiglas box featuring a grid floor (3 mm stainless steel rods set 5 mm apart) with a wooden platform (7 × 7 × 1.7 cm^3^) in the center of the grid floor. In training session, each mouse was gently placed on the wooden platform set in the center of the grid floor. When the mouse stepped down and placed four paws on the grid floor, a 36 V shock was delivered for 2 s and step-down latency was recorded. Tests were taken 24 h after training; each mouse was again placed on the platform, and the latency was recorded with an upper cut-off time of 180 s.

### 2.4. Morris Water-Maze Task

The procedure of Morris water maze (MWM) was described previously [33]. Briefly, a plastic platform (diameter: 10 cm; height: 30 cm) was placed at the center of one quadrant in a pool with a diameter of 100 cm and height of 40 cm. Before the experiment, the pool was filled with sufficient water so that the platform was approximately 1-2 cm beneath the water surface, and the water temperature was fixed at 22 ± 1°C. During the experiment, all objects in the room were fixed in place to provide additional cues to enable the animals to locate the platform. Each animal was subjected to 4 trials per day for 6 consecutive days. After 6 days of training, the platform was removed from the pool and each animal was then placed in the pool at the same position and was allowed to swim for 1 minute. The swim velocity, latency in finding the platform, and time in the target quadrant were analyzed using the Any-maze. After 1 minute, the animal was removed from the maze, dried with a towel, and returned to its cage beside an electric radiator.

### 2.5. E2 Radioimmunoassay

Trunk blood was collected and the plasma was stored at −30°C until assayed. Plasma levels of E2 were quantified by an ultrasensitive radioimmunoassay. E2 were assayed using commercially available RIA kits (Shanghai Institute of Biological Product, Shanghai, China). The mean intra- and interassay coefficients of variation for E2 were 5.78% and 6.96%, respectively.

### 2.6. Measurement of Glycine NO

Measurements of the stable end products of NO, nitrite and nitrate, provide a qualitative measure of NOS activity and NO production [34–36]. Nitrite and nitrate were determined following the reduction of nitrate to nitrite using nitrate reductase and the NADPH regenerating system (G-6-P/G-6-PDH) as described previously [37]. In brief, samples were incubated with reaction mixture (nitrate reductase 30 mU; NADPH 3 *μ*M; G-6-P 750 *μ*M; G-6-PDH 48 mU in a final reaction volume of 100 *μ*L) for 90 min at room temperature in a 96-well microtiter plate. At the end of incubation, 30 *μ*L of 0.62 N HCl and after 10 min 30 *μ*L of 1.4 N NaOH were added to the incubation mixture. The fluorescence was measured at *λ*ex 360 nm and *λ*em 450 nm using a microtiter plate reader (PerkinElmer, USA). Tissue NO levels were expressed as nmoles/mg of cytosolic protein, and plasma NO levels were expressed as *μ*M.

### 2.7. Measurement of Glycine

Amino acids levels were measured by using high-performance liquid chromatography (HPLC) as previously reported [38]. Briefly, the brain tissues were homogenized in 20 volumes of methanol on ice. The homogenates were centrifuged at 4500 g for 10 min, and 20 *μ*L of supernatant was evaporated to dryness at 40°C. To the residue, 20 *μ*L of water (H_2_O), 20 *μ*L of 0.1 M borate buffer (pH 8.0), and 60 *μ*L of 50 mM 4-fluoro-7-nitro-2,1,3-benzoxadiazole (NBD-F) in acetonitrile (CH_3_CN) were added. The reaction mixture was then heated at 60°C for 1 min and immediately supplemented with 100 *μ*L of H_2_O/CH_3_CN (90/10) containing 1% trifluoroacetic acid to stop the reaction. Ten microliters of the resultant solution was injected into the HPLC system.

### 2.8. Statistical Analysis

All data are expressed as mean ± SEM. Origin 7.5 (Originlab Co., USA) and SigmaStat 3.5 (Systat Software, Inc, USA) were used to plot and analyze data by Student's *t*-test for 2 groups, and one-way analysis of variance (ANOVA) was used for >2 groups, followed by a Student-Newman-Keuls (SNK) post hoc test; the escape latency during the training sessions of MWM test was analyzed by two-way repeated-measures ANOVA followed by a SNK post hoc test. *P* < 0.05 was taken as statistically significant.

## 3. Results

### 3.1. Effects of DSS on Step-Down Test

**Figure 1:**
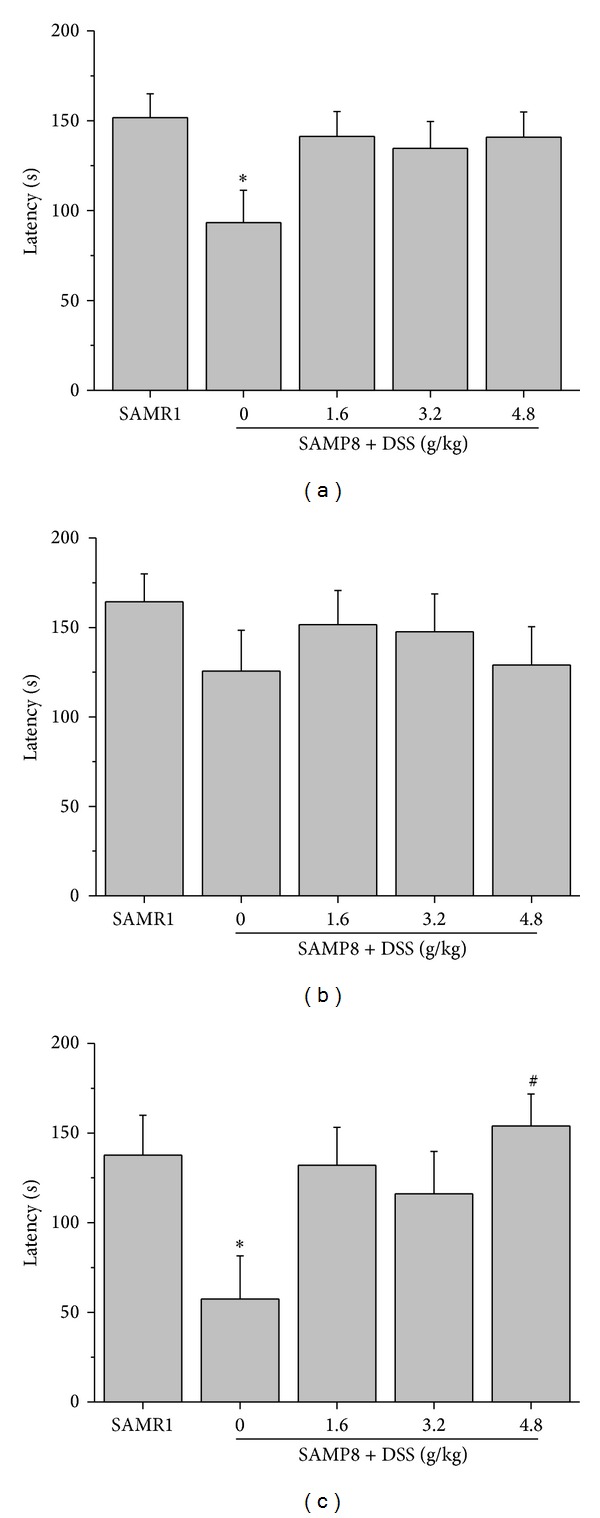
Effects of DSS on step-down test. Effects of DSS on passive avoidance ability during the step-down test in SAMP8 for all animals (male and female SAMP8, *n* = 19–20) (a), male animals (*n* = 9-10) (b), and female animals (*n* = 9-10) (c); *P < 0.05, compared with SAMR1; ^#^ *P* < 0.05, compared with SAMP8. The data are expressed as the mean ± SEM.

In the step-down test, the latency of SAMP8 is shorter than age-matched SAMR1, especially in female animals. DSS increased the latency in female SAMP8 significantly at the dose of 4.8 g/kg (Figures 1(a), 1(b), and 1(c)).

### 3.2. Effects of DSS on MWM Test

**Figure 2:**
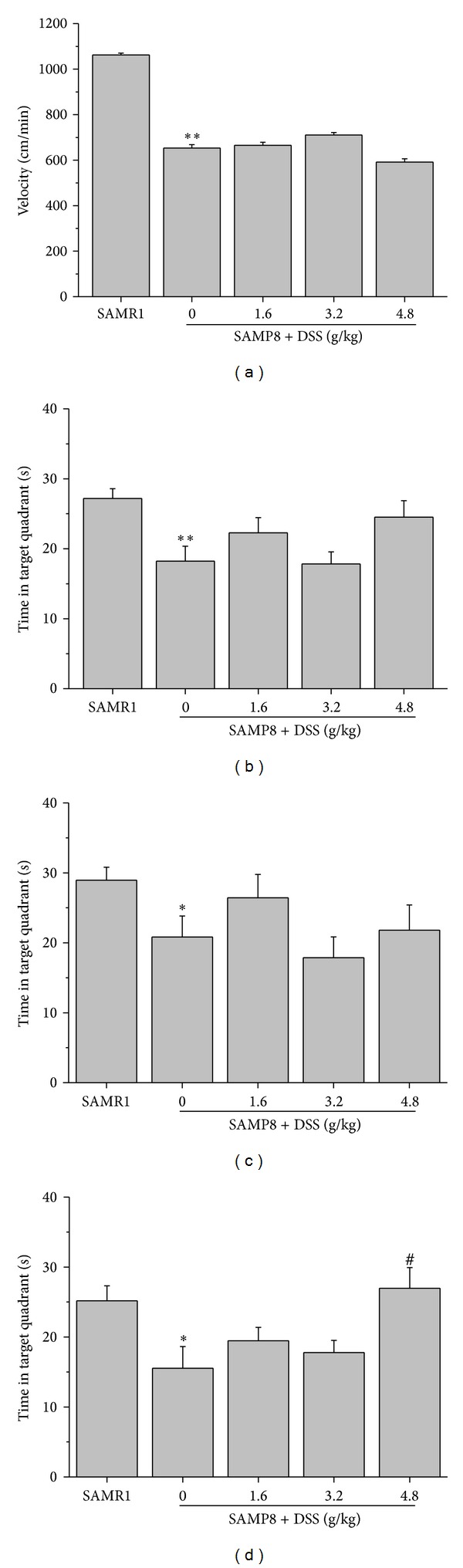
Effects of DSS on MWM test. Velocity of SAMR1 and SAMP8 during the test sessions of the Morris water maze performance (male and female, *n* = 19-20) (a), time spent in the target quadrant during test session for all animals (male and female SAMP8, *n* = 19-20) (b), male animals (*n* = 9-10) (c), and female animals (*n* = 9-10) (d); *P < 0.05, **P < 0.01, compared with SAMR1;^ #^ *P* < 0.05, compared with SAMP8. Data values are expressed as mean ± SEM.

In the Morris water maze performance training sessions, SAMP8 took more time to find the platform compared with SAMR1. DSS shortened the latency significantly, especially for female SAMP8 (Tables 1, 2, and 3). Meanwhile, SAMP8 swam more slowly than SAMR1, and DSS has little effect on the swim velocity of SAMP8 (Figure 2(a)).

On the day of probe trial following the final day of training trial, SAMP8 spent less time in the quadrant, where former platform was placed, than SAMR1; DSS increased the time spent by SAMP8 in the target quadrant, especially in female animals (Figures 2(b), 2(c), and 2(d)).

### 3.3. Effects of DSS on E2 in Blood Plasma

**Figure 3:**
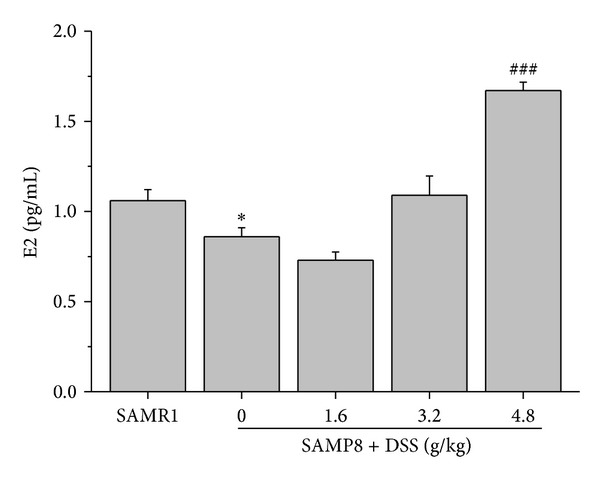
Effects of DSS on E2 in blood plasma. The effects of DSS on the plasma E2 concentration in female SAMP8. *P < 0.05, compared with SAMR1; ^###^ *P* < 0.001, compared with SAMP8; data are expressed as mean ± SEM, *n* = 6–10.

Compared with SAMR1, E2 level was decreased in SAMP8, and DSS elevated the plasma E2 in female SAMP8 significantly (Figure 3).

### 3.4. Effects of DSS on NO in the Blood Plasma and Hippocampal Tissue

**Figure 4:**
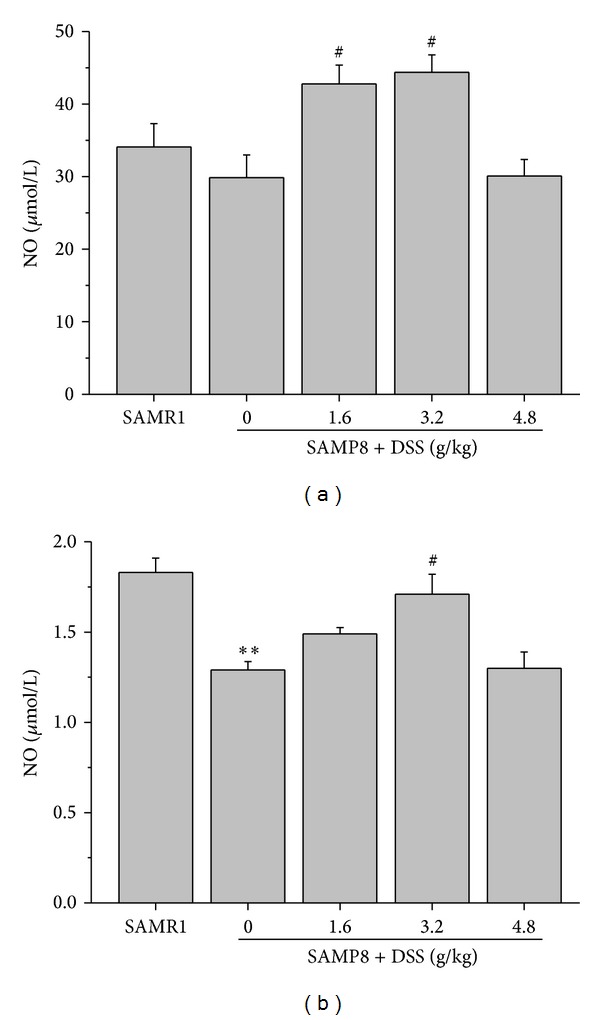
Effects of DSS on NO in the blood plasma and hippocampal tissue. The effect of DSS on the NO level in plasma (a) and hippocampus (b) of female SAMP8. **P < 0.01, compared with SAMR1; ^#^ *P* < 0.05, compared with SAMP8; data values are expressed as mean ± SEM, *n* = 9-10.

Compared with SAMR1, NO level was lower in both plasma and hippocampus tissue in SAMP8, especially in hippocampus. DSS elevated NO level both in blood plasma and hippocampus tissue (Figure 4).

### 3.5. Effects of DSS on Glycine in Hippocampal Tissue

**Figure 5:**
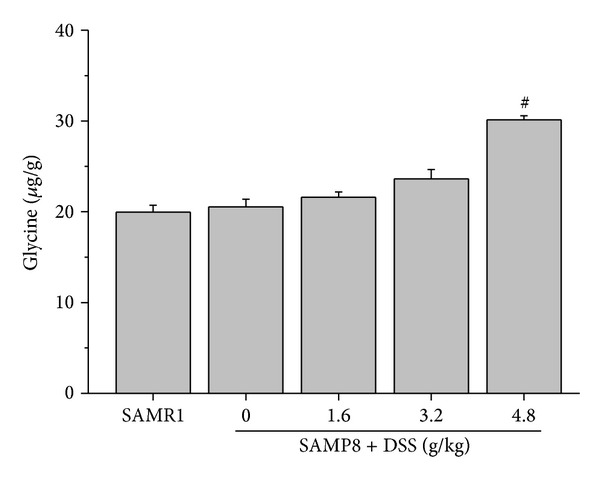
Effects of DSS on glycine in hippocampal tissue. The effect of DSS on the glycine level in hippocampus of female SAMP8. ^#^ *P* < 0.05, compared with SAMP8; data values are expressed as mean ± SEM, *n* = 9-10.

There is no difference of glycine in hippocampal tissue between SAMP8 and SAMR1. DSS increased the glycine level in SAMP8 significantly (Figure 5).

## 4. Discussion

SAM was originally developed from the AKR/J strain mice in 1968 in the laboratory of Professor Toshio Takeda in Kyoto, Japan, based on the data of the grading score of senescence, life span, and pathologic phenotypes [39, 40]. SAMP8 is characterized by early onset of deficits in learning and memory, cholinergic deficit in the hippocampus, age-related increase in A*β*-like deposition, and amyloid plaques [41–43]. A comparison of the properties of SAMP8 and the characteristic features of AD shows some similarities, suggesting that SAMP8 serves as a good animal model to investigate the fundamental mechanisms of AD and assess the action of drugs [44, 45]. In this study, we found that plasma E2 is decreased in female SAMP8 compared with female SAMR1, and the cognitive ability also declined greater in female SAMP8 than males. These results indicated that female SAMP8 could be considered as an animal model for female AD patients.

Deposition of amyloid-beta (A*β*) in the brain is believed to be the critical step at AD onset [46]; the ability of estrogens to reduce A*β* accumulation may be their most important neuroprotective action against AD [47, 48]. E2 can increase the nonamyloidogenic pathway by promoting the production of *α*-APPs and, as a consequence, can reduce the amount of A*β* generated [49–53]. In addition, estrogens could regulate other processes against AD, including spine density [54], long-term potentiation [55], neurotransmitter systems [56], protection against neuron cell death [57, 58], and tau hyperphosphorylation [48]. After menopause, the level of estrogens dropped sharply, and this caused women to be more vulnerable to AD. Many observational and clinical trials in human suggested that hormone treatment is associated with reduced incidence of AD [59–63]. So the increasing of estrogen in female SAMP8 might be one of the mechanisms for cognition enhancement effects of DSS.

Nitric oxide (NO) liberated from postsynaptic neurons may travel back to presynaptic terminals to cause LTP expression [64] and play an important role in synaptic transmission. In AD patient, eNOS and NFTs and SPs have a significant negative correlation [65]. Estrogens could induce NO production via estrogen receptors (ERs) [66]. In this study, we found that DSS could increase the content of NO in the hippocampal tissue of SAMP8 at dose of 1.6 g/kg and 3.2 g/kg, so modulation of NO might be another mechanism for DSS against AD. It is unclear why DSS has no effect on the level of NO at the dose of 4.8 g/kg. Some materials contained in DSS might inhibit NO production. It is reported that Paeoniflorin, a small molecular compound in DSS, could inhibit NO level in some experimental conditions [67, 68].

NMDA receptors are essential for cognitive abilities. In 1986, Morris reports the first evidence that NMDA receptors are necessary for spatial learning [69]. These results were confirmed by another study showing that a knock-out of the NMDA receptor in CA1 results in deficits in LTP and spatial memory [70]. Recently, many reports have indicated that the function of NMDA receptors decreases in AD, including gene expression [71], neurotransmitters (such as glutamate) [72], and coactivator of NMDA receptor (such as D-serine) [73]. Deficits in glutamatergic system were also observed in animal models, such as senescence-accelerated mouse/prone 8 (SAMP8) [74, 75]. Glycine is a coagonist of NMDA receptors, and increasing glycine concentration in the synaptic cleft can improve cognitive impairment in animal models of AD [76] indicating that increasing glycine is beneficial for AD. A previous study showed that DSS could elevate glycine in female SAMP8 [77], and we got the same results in this study. Estrogen was proved to stimulate glycine incorporation [78], and increasing glycine in hippocampal tissue might contribute to the protective effects for DSS against AD.

**Figure 6:**
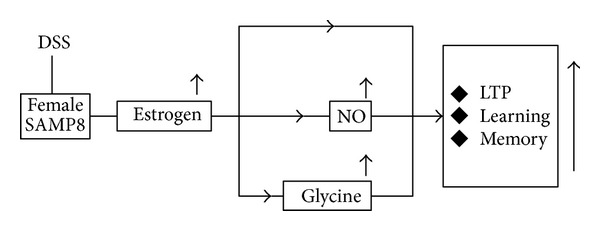
Proposed mechanisms of DSS on cognition improving effects in female SAMP8.

In conclusion, DSS has better effects on female SAMP8 than males. Plasma E2 in female SAMP8 was increased by DSS in a dose dependent manner. These results have good consistency with our hypothesis, indicating that DSS might be more effective in female patients than males. DSS plays its protective role against AD via modulation of estrogen, NO, and glycine in plasma or hippocampal tissue (Figure 6).

## Figures and Tables

**Table 1:** Effects of DSS on the latency during the training trial sessions for all animals (male and female SAMP8).

Day	Escape latency (s)				
	SAMR1	SAMP8	SAMP8 + DSS (g/kg)		
			1.6	3.2	4.8
1	28.65 ± 2.38	39.14 ± 3.41	35.56 ± 2.60	32.96 ± 2.87	37.24 ± 3.12
2	22.40 ± 3.04	40.52 ± 3.73**	30.26 ± 3.61	31.85 ± 2.58	29.86 ± 3.61^#^
3	21.84 ± 2.96	38.97 ± 2.98**	33.00 ± 3.42	29.77 ± 2.96^#^	32.03 ± 4.53
4	14.33 ± 1.96	33.85 ± 3.80**	30.97 ± 3.36	32.89 ± 3.31	27.90 ± 4.02
5	11.62 ± 1.35	29.88 ± 3.63**	22.20 ± 3.44	21.10 ± 1.84^#^	28.72 ± 4.21
6	10.67 ± 1.77	30.02 ± 4.06**	23.76 ± 3.46	20.88 ± 2.55	28.64 ± 4.48

**P < 0.01, compared with SAMR1; ^#^ *P* < 0.05, compared with SAMP8. Data values are expressed as mean ± SEM, *n* = 19-20.

**Table 2:** Effects of DSS on the latency during the training trial sessions for male SAMP8.

Day	Escape latency (s)				
	SAMR1	SAMP8	SAMP8 + DSS (g/kg)		
			1.6	3.2	4.8
1	30.53 ± 2.35	38.66 ± 5.48	37.43 ± 3.57	32.03 ± 4.77	37.13 ± 5.31
2	24.01 ± 4.78	35.94 ± 5.40	30.56 ± 5.73	33.53 ± 3.39	33.61 ± 5.03
3	21.18 ± 4.37	34.68 ± 3.93	35.68 ± 5.80	34.60 ± 3.97	36.92 ± 6.76
4	16.47 ± 3.95	30.27 ± 4.64*	26.38 ± 4.40	37.42 ± 4.63	32.89 ± 6.07
5	13.04 ± 2.16	24.35 ± 3.25**	17.73 ± 5.19	20.43 ± 3.03	34.16 ± 5.91
6	13.01 ± 3.09	23.55 ± 4.45	26.51 ± 5.82	22.93 ± 4.30	36.65 ± 7.11

*P < 0.05, **P < 0.01, compared with SAMR1; data values are expressed as mean ± SEM, *n* = 9-10.

**Table 3:** Effects of DSS on the latency during the training trial sessions for female SAMP8.

Day	Escape latency (s)				
	SAMR1	SAMP8	SAMP8 + DSS (g/kg)		
			1.6	3.2	4.8
1	26.57 ± 4.35	39.66 ± 4.19*	33.70 ± 3.68	33.90 ± 3.17	37.36 ± 3.32
2	20.62 ± 3.80	45.61 ± 4.84**	29.96 ± 4.41^#^	29.99 ± 4.06^#^	25.70 ± 5.11^#^
3	19.25 ± 4.03	43.73 ± 4.17**	30.32 ± 3.41^#^	24.39 ± 3.88^##^	26.60 ± 5.76^##^
4	11.95 ± 2.45	37.83 ± 6.17**	35.55 ± 4.61	27.86 ± 4.38	22.35 ± 4.84
5	10.04 ± 1.48	36.02 ± 6.37**	26.67 ± 4.01	21.84 ± 2.13	22.67 ± 5.63
6	8.06 ± 1.14	38.10 ± 6.44**	21.00 ± 3.52^#^	18.61 ± 2.57^#^	19.75 ± 3.67^#^

*P < 0.05, **P < 0.01, compared with SAMR1; ^#^ *P* < 0.05. ^##^ *P* < 0.01 compared with SAMP8. Data values are expressed as mean ± SEM, *n* = 9-10.
